# A Phenotypic Approach to the Discovery of Potent G-Quadruplex Targeted Drugs

**DOI:** 10.3390/molecules29153653

**Published:** 2024-08-01

**Authors:** Stephen Neidle

**Affiliations:** The School of Pharmacy, University College London, London WC1N 1AX, UK; s.neidle@ucl.ac.uk

**Keywords:** quadruplex DNA, pancreatic cancer, phenotypic screening, QN-302, structure-based design

## Abstract

G-quadruplex (G4) sequences, which can fold into higher-order G4 structures, are abundant in the human genome and are over-represented in the promoter regions of many genes involved in human cancer initiation, progression, and metastasis. They are plausible targets for G4-binding small molecules, which would, in the case of promoter G4s, result in the transcriptional downregulation of these genes. However, structural information is currently available on only a very small number of G4s and their ligand complexes. This limitation, coupled with the currently restricted information on the G4-containing genes involved in most complex human cancers, has led to the development of a phenotypic-led approach to G4 ligand drug discovery. This approach was illustrated by the discovery of several generations of tri- and tetra-substituted naphthalene diimide (ND) ligands that were found to show potent growth inhibition in pancreatic cancer cell lines and are active in in vivo models for this hard-to-treat disease. The cycles of discovery have culminated in a highly potent tetra-substituted ND derivative, QN-302, which is currently being evaluated in a Phase 1 clinical trial. The major genes whose expression has been down-regulated by QN-302 are presented here: all contain G4 propensity and have been found to be up-regulated in human pancreatic cancer. Some of these genes are also upregulated in other human cancers, supporting the hypothesis that QN-302 is a pan-G4 drug of potential utility beyond pancreatic cancer.

## 1. Introduction

There is considerable current interest, notably in the academic community [1,2,3,4,5], in directly targeting the higher-order DNA motifs (G-quadruplexes: G4s) in those genes which are relevant to human cancers using drug-like small molecules. The goal is to selectively interfere with the processes of transcription, translation, or replication in cancer cells and thus inhibit tumor growth. Two such compounds have entered clinical evaluation for human cancers, CX-5461 (Pidnarulex) [6,7,8] and QN-302 [9,10], which can be classified as experimental drugs rather than solely as G4-binding agents. 

The focus of this article is to present the background and the distinctive approach to the discovery and development of one of these drugs (QN-302) as an anticancer agent. We also describe the plausible gene targets for QN-302 that have emerged to date from whole-genome transcriptome studies. There is increasing interest in targeting G4s in viruses, bacteria, and parasitic targets [11,12,13,14,15,16]. Although plausible small molecule G4 ligands have been identified for some of these, none have yet reached a clinical assessment stage and they will not be discussed further here, although the methodology described below may also be applicable in these fields. 

G-quadruplexes are higher-order DNA (and RNA) structures formed by the association of successive short G-tracts [17,18,19,20,21] and have characteristic four-guanine Hoogsteen hydrogen-bonded planar motifs, termed G-quartets (or tetrads). The consequence of G-quartet formation in single-stranded G-tract sequences is that the backbone of intramolecular G4s is folded back at least three times, giving rise to the “four-stranded” nature of G4 structures, in contrast to the two anti-parallel strands of canonical duplex DNA. G4s require metal ions for stability, most often K^+^ or Na^+^, which sit in the central channel of a G4, either in the plane of a G-quartet or between two adjacent ones, in close electrostatic contact with the O6 atoms of the guanine bases forming the G-quartets.

The majority of G4s encoded in the human genome usually comprise four G-tracts, and are typified by the general sequence:

…….GaXnGbXoGcXpGd……

where a…d are the numbers of G residues in each short G-tract, typically 2–4, and are usually directly involved in G-quartet formation [18,21,22,23].

The sequences linking the G-tracts, Xn, Xo, and Xp, can be any combination of residues, including G, which form loops in these right-handed G4 structures [24]. This notation also suggests that the G-tracts can be of unequal length, and if this is the case, then some of the G residues can be part of loop regions. The assumption that all G-tracts within a quadruplex sequence are identical is true for vertebrate telomeric sequences but is frequently not the case for non-telomeric human genomic sequences, or even for all telomeric sequences in some lower eukaryotic organisms. G4s can have a variety of topologies, varying in mutual strand direction and depending on such factors as loop size and character [18,21,22,24]. Some G4s are highly polymorphic, typified by those formed by repeats of the human telomeric DNA sequence. Various G4s with all-parallel, hybrid, and all-anti-parallel strands have been characterized. The major right-handed simple G4 topologies determined to date are defined by their backbone directionality; these are parallel, antiparallel (chair and basket), and hybrid forms 1 and 2, all of which have been observed in human telomeric G4s [25,26]. The cytosine-rich strand complementary to a G4 sequence can also form distinct four-stranded structures, termed i-motifs [27], which, even though they are less stable than G4s at physiological pH, may also have discrete biological functions [27,28].

G4s are non-randomly distributed in the human genome. Initial bioinformatics estimates based on the above general sequence suggested a total occurrence of ca 376,000 unimolecular G4s [22,23], not all of which occur within genes. Subsequent relaxation of the sequence limits to include potentially longer loops than the originally assumed seven-nucleotide maximum [22,23] increases this to 2-–10-fold more putative and potentially stable G4s [29,30] than the original estimates, although the exact numbers predicted depend on the algorithm used and the underlying assumptions about the sequence [30]. The advent of G4-specific antibodies [31] combined with next-generation sequencing methodology has enabled direct experimental assessments of G4 numbers in cells to be made and their occurrence to be mapped. Some 10,000 G4 occurrences have been mapped in a human breast cancer cell line, compared with ca 1500 in a normal cell line [32], suggesting that G4s may be useful targets for cancer cell selectivity and possible therapeutic intervention. G4s sometimes occur in clusters, implying added structural and biological complexity.

G4s are over-represented in the promoter regions of many cancer-linked genes [33,34,35,36,37] as well as in 5′- and 3′-untranslated regions [38,39,40]. Their presence in individual promoter sequences, normally close to transcription start sites (for example in the oncogenes *KRAS* [40,41,42], *c-KIT* [43,44], *MYC* [45,46,47,48]), has been of particular interest. Many studies have indicated that the binding of a G4-selective small molecule can inhibit the transcription of these genes (see for example references [42,44,45,47,48]), possibly by hindering transcription factor interaction with the promoter and the G4. To date, with a few exceptions, targeting RNA G4s has not been of comparable focus, although their potential structural complexity suggests that future efforts in this area may be fruitful (see for example [49,50,51]).

Table 1 lists the human promoter G4 molecular structures determined to date by crystallography and/or NMR methods. All these G4s have a cancer association, mostly by being within established oncogenes. The small size of this list (14 entries) is remarkable, given that the number of known mutation-driven cancer genes is at least 727 [52], and that many more genes are known to be dis-regulated, especially in common solid cancers such as breast, pancreatic, and prostate cancer (see Section 3 below). G4s appear to have multiple functions [53] in addition to their involvement in transcriptional initiation, depending on their location in the human genome and in individual genes. They have been extensively implicated in replication [54], translation [38,39], DNA damage [55,56], genome rearrangements [57,58], and genome instability [59]. The effects of G4 ligands on these processes is yet to be thoroughly explored, in contrast with the emphasis to date on G4 promoter–ligand binding.

G4s within the promoter of the common oncogene *MYC* have been shown to play a major role in the transcription of this gene, with these promoter G4s being direct targets for transcription factors such as SP1 [60]. One can speculate that the formation of a G4-ligand complex results in steric hindrance to G4 accessibility, in a way yet to be determined, so that transcription factor binding is no longer achievable. This is consistent with G4 end-capping by ligands, observed experimentally in G4-ligand structures (ligand capping may also be involved in the mechanism of action of telomeric G4-targeting agents—see below). It has been suggested that the G4 promoter regions in multiple cancer genes need to be simultaneous targets for those complex cancers with multiple genetic aberrations. Multiple G4 targeting may thus have potential for more fruitful therapeutic outcomes than single-gene targeting [61,62,63,64]. To date, there have been rather few studies that have examined this issue in detail; instead, in most instances, a correlation between a single gene’s downregulation and potent ligand binding to its promoter G4 has been taken to demonstrate a direct mechanistic link. This may be the case in many instances (see Section 3c). However, this is not necessarily universally true, as shown by a careful study [65] involving *MYC* targeting that has clearly revealed that indirect effects can occur. These can deceive one into accepting at face value a correlation between G4s and the downregulation of gene expression as direct cause and effect, adding complexity to the seductively simple concept of promoter G4 targeting. On the other hand, some unequivocal demonstrations of a direct link between gene downregulation and G4 action have been achieved, for example with *MYC* and the ellipticine derivative GQC-05 using a pair of cell lines with exon-specific exploitable differences [66]. 

### 1.1. Native G4 Structures

The topology and geometry of many G4s have been determined by X-ray crystallographic and NMR methods [21], although, as stated above, few are of promoter G4s. Structural data and relevant information are available in the Protein Data Bank (the PDB: www.rcsb.org), the more detailed Nucleic Acid Database (www.nakb.org, last accessed on 15 July 2024), and the specialized G4 database ONQUADRO (https://onquadro.cs.put.poznan.pl/, last accessed on 9 July 2024). G4 prediction programs are useful tools for evaluating the propensity of a given sequence to form a G4. QGRS Mapper (https://bioinformatics.ramapo.edu/QGRS/, last accessed on 3 May 2024) and G4 Hunter (http://bioinformatics.ibp.cz, last accessed on 3 May 2024) are widely used, especially in conjunction with biophysical methods such as circular dichroism and melting profiles, to determine G4 formation and stability. There are also a growing number of G4-protein structures, with their details similarly available in the PDB.

Almost all the native G4 structures deposited to date are of discrete DNA G4s formed by folding G4 sequences. Many of the deposited structures are of telomeric G4s, from human and other organisms, with small changes in the G4 or flanking sequence often resulting in topological change. These are equivalent to (sometimes with altered 5′ or 3′ nucleotides), and sometimes identical to, the sequences used in biophysical and ligand-binding studies.

G4s share the common feature of a core comprising stacked G-quartets, held together by backbones and loops. The variety of G4 structures mostly reflects differences in their loop size, sequence, number of G-quartets, and strand polarity. This variety is also manifest in size differences between G4 grooves [67]. B-form duplex DNA has a well-defined major and a minor groove, with the latter having a sequence-dependent spine of hydration. High-resolution G4 crystal structures have also identified discrete structured water molecule spines within the grooves, which can be assumed to play a role in small molecule and protein binding [67,68].

There are currently no reported structural studies of a DNA G4 embedded in its fully natural genomic duplex environment. However, a recent cryo-electron microscopy analysis at a resolution of 7.4 Å [69] of a G4 embedded between two DNA duplexes comes close to being the sole exception to date (PDB id 8DU). The G4 in this more complex structure is from the *MYC* promoter NHEIII (nuclease-hypersensitive element III) sequence. The two duplexes are not co-linear as previously assumed but are oriented at an angle of 53° to each other, with the G4 and a region of unstacked duplex in between them. The complementary strand to the G4 in this structure is not a natural C-rich i-motif one, but a synthetic poly T loop construct. Even so, the structure reveals that the central G4 has potentially accessible terminal G-quartet surfaces together with the adjacent and partially unwound duplex region. These surfaces are then analogous to those observed in the crystal and NMR structures of isolated G4s (Table 1) and may be available for small molecule binding. Other crystal and NMR structures of G4s with a duplex at one end have been observed [70,71] and similarly reveal an accessible planar terminal G-quartet at an end of the G4. This feature appears to be a conserved and biologically relevant general recognition motif for small molecule binding, with potential for specificity arising from non-bonded interactions with the surfaces and clefts in the duplex–G4 junctions.

### 1.2. Structures of G4 Small Molecule Complexes

The molecular structures of many low-molecular weight compounds with isolated G4s have been determined by crystallography and NMR. A selection of these is listed in Table 2. The overwhelming majority involve human telomeric G4s (see below). The ligands are generally characterized by having a planar extended chromophore with from one to four appended cationic side chains. A few do not conform to this general chemotype, having for example a macrocyclic core, as in the natural product telomestatin [72,73]. By contrast, the synthetic compound pyridostatin has a single central pyridine ring at the center of the molecule, with two attached side-arms, each culminating in a substituted quinoline group [55,74].

Regardless of the nature of the G4 ligand, their consistent binding mode has the chromophore, macrocycle, or central heteroaromatic ring stacked and stabilized by π–π stacking interactions with a terminal G-quartet at one end of the target G4. Side chains occupy grooves, although the crystal and NMR structures show that they rarely completely occupy groove space. An examination of several high-resolution crystallographic analyses has shown that conserved water molecules, embedded in the grooves, play a role in mediating side-chain binding between ligand and hydrogen bond donors/acceptors on the groove surfaces [68].

A microarray approach has been used to identify a novel small-molecule compound (DC-34) that binds specifically to a *MYC* promoter G4. This compound does not conform to the conventional view of a G4 ligand [75,76]. It does not have an extended aromatic chromophore or extended cationic side-arms. The associated NMR structure of the G4 complex [76] has shown that, perhaps surprisingly, the benzofuran and methylbenzene rings of DC-34 stack on both terminal G-quartets of the *MYC* G4, forming a 2:1 ligand:G4 complex. It was also definitely demonstrated that DC-34 selectively down-regulates *MYC* expression via its G4 binding and has a minimal effect on a panel of other G4-containing oncogenes such as *BCL2* and *KRAS*.

## 2. The Targeting of Human Telomeric G4s

Human chromosomal ends, termed telomeres, comprise the DNA sequence TTAGGG, which is repeated several thousand times, together with associated proteins [77]. Although most telomeric DNA exists naturally in a standard Watson–Crick duplex form, the extreme ends of ca 100–200 nucleotides are single stranded. These would naturally fold into G4 structures, typically with 3–4 repeats [78,79], but are hindered in doing so by specific telomeric single-stranded DNA binding proteins, such as hPOT1 [80]. Human telomeric DNA progressively shortens during successive rounds of replication, until it reaches a critical point (the Hayflick limit) when cells enter a senescent stage as a prelude to apoptosis [81]. However, over 80% of tumor cells have evolved a way to circumvent this and become immortalized. This is achieved in most cancer cells by expression of the reverse transcriptase enzyme telomerase, which progressively synthesizes TTAGGG repeats onto the 3′ ends of telomeric DNA, maintaining its length [82]. Conversely, telomerase activity can be inhibited by inducing the folding of the single-stranded ends into G4s, since the synthesis of a TTAGGG repeat on the telomerase template requires the telomere end substrate to be single-stranded [83]. The consequence of telomerase inhibition is eventual senescence and cell death for an affected cancer cell. It was subsequently hypothesized [84] that the G4 folding of telomeric DNA ends could be facilitated by using a G4-selective small molecule to stabilize 3′-terminal G4s. A library of G4-binding disubstituted amido-anthraquinones (AQs) was shown to do just this, resulting in measurable telomerase inhibition [84]. This was the first study to show that small-molecule binding to a G4 could have a biological (and a potential therapeutic) effect. This working hypothesis used the very limited G4 structural data available at the time to suggest that the two cationic side chains of these AQ ligands would effectively occupy two grooves of the NMR structure of a human intramolecular telomeric G4, which was taken as a plausible model [85].

**Figure 1:**
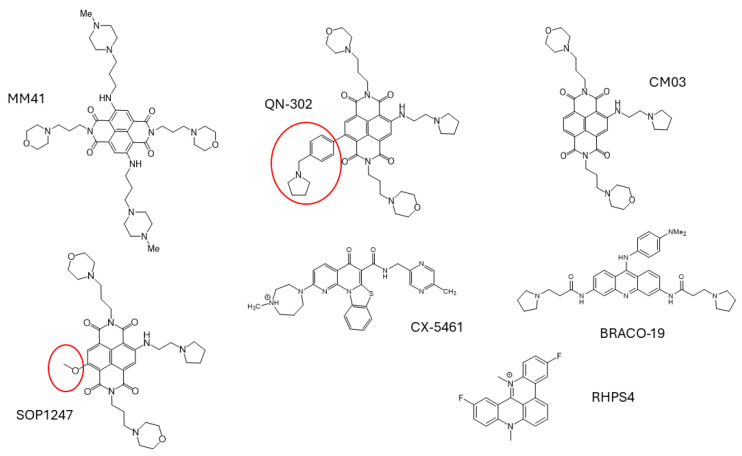
Structures of G4 ligands and experimental drugs discussed in this text. The substituent group in QN-302 responsible for enhanced potency is circled in red, as is the methoxy group in the short-listed ND lead compound SOP1247.

Subsequent studies revealed the onset of cellular senescence and apoptosis following ligand-induced telomerase inhibition by AQs and other G4 targeting compounds (see for example references [86,87,88]). In a few instances, in vivo anti-tumor effects were observed in cancer xenograft models, for example with the tri-substituted acridine compound BRACO-19 (Figure 1) [89]. This compound had been designed [90] by qualitative molecular modelling to have superior G4 vs duplex selectivity compared to the disubstituted AQ ligands and thus had an improved telomerase potency. It was hypothesized that the three side chains of BRACO-19 would each bind in a G4 groove, driving improved G4 binding, telomerase potency, and G4 selectivity. These predicted changes were observed. The modelling used the (then) sole human telomeric G4 experimental structure available, the anti-parallel structure in Na^+^ solution, determined by NMR [85]. This structural concept was subsequently validated by an X-ray crystallographic study of BRACO-19 bound to a human telomeric bimolecular G4 [91], which also showed that BRAC-19 conforms to the standard model of ligand–G4 interaction, i.e., its acridine chromophore end-stacks onto a G4. Many synthetic compounds and natural products, notably the cyclic natural product telomestatin and derivatives of BRACO-19, have been evaluated as potential anti-tumor agents [87,92,93]. They have been presumed to act via a telomerase-mediated mechanism, although few have reached the point of detailed in vivo mechanistic evaluation, in part due to pharmacological challenges.

Telomerase targeting via G4s was eventually abandoned as an anticancer approach, in part because: (1) compounds such as the trisubstituted acridines only showed moderate anti-tumor potency at best [94], and (2) some, such as the pentacyclic acridines, typified by the compound RHPS4 [95], had significant off-target cardiotoxic liabilities (hERG), which an analogue campaign failed to eliminate [96,97]. There was a view at the time that all G4 ligands would have inherent hERG or neuro-receptor off-target toxicity. Subsequent detailed receptor binding studies on later chemical series such as the naphthalene diimides have shown that this view is erroneous. However, as in any serious drug discovery program, it is vital to check for such effects and not shy away from making the decision to abandon a particular chemotype if it is associated with serious adverse effects, especially those that are manifest at doses close to therapeutic ones.

In addition, the mechanism of action of these compounds is more complex than suggested by the simple model outlined above. It is now apparent that compounds such as BRACO-19 and RHPS4 act, at least in part, not by G4-directed telomerase inhibition but instead by telomere end-uncapping [89,97]. This involves initial G4-ligand formation competing with single-strand binding proteins, resulting in the exposure of telomeric DNA ends. This event triggers DNA damage responses [55,56,98]. Such a mechanism is unlikely to be cancer cell selective, in accordance with the modest in vivo anticancer activity shown by these G4 ligands.

However, the telomerase inhibition/telomere uncapping approach has recently received renewed attention with several promising studies, suggesting that, with appropriate G4 ligands, it may be worth further investigation [99,100,101].

## 3. Promoter G4s as Therapeutic Targets

The seminal study by Hurley et al. [45] demonstrated that the porphyrin ligand TMPyP4 could down-regulate the expression of the *MYC* oncogene via promoter G4 binding. This work has been followed (see for example references [34,35,36,37]) by very many studies in which a given promoter G4 has been targeted by a G4 ligand, with readouts of high G4 affinity and gene downregulation, most commonly from biophysical studies together with an in vitro expression assay. Sometimes expression changes are assessed in cellular experiments (see for example references [44,47,48,65,102,103,104,105,106]). Popular target cancer-relevant genes include *MYC*, *KRAS*, *c-KIT*, *BCL-2*, and *hTERT* [34,35,36,37]. However, this approach currently has several challenges:

- **The overwhelming majority of known G4 structures have broadly similar small-molecule recognition features.** These are: (1) the planar terminal G-quartets at the ends of a G4, and (2) multiple grooves/loops, albeit of differing sizes and electrostatic charge/hydrogen bond donor/acceptor distribution, depending on the G4 topology and sequence. The recognition challenge involves the selection of a single G4 in a single gene in a cell by a typical small molecule ligand from the set of ca 10,000 G4s encoded in the transcriptionally active genes in a cancer cell [32] (see the G4 ligand database https://www.g4ldb.com/ with over 4800 entries, last accessed 25 July 2024).

Thus, the cellular selection of a single gene G4 from the global G4 gene pool is currently implausible. However, some progress has been made, in the case of the *MYC* gene, using a microarray approach [75,76]. One caveat is that the selectivity of the small molecule DC34 derived from this study was evaluated on a small panel of G4s and not on the complete genome.

Structure-based design approaches have also been extensively reported but, at the present state of knowledge, are unlikely to succeed in identifying a compound capable of single-gene G4 selection given the above challenges. In addition, the currently available data set of promoter G4 molecular structures (Table 1) is very small. The added structural complexity of a G4 fully embedded in a duplex environment is indicated by the sole (low-resolution) currently available structure [69] that shows a G4 in a more biologically relevant context. It is hoped that future higher resolution structures of promoter G4–duplex complexes will become available and will highlight features exploitable for ligand specificity. These are not apparent in the current G4 structures that lack their duplex context.

Existing G4 structures have the greatest variability in terms of the size and nature of their grooves and loops, with longer loops such as in the *hTERT* promoter G4 being themselves capable of secondary structure formation, adding to potential selectivity. Several studies (see for example references [107,108,109,110]) have employed in silico screening to find drug-like G4-binding ligands, sometimes locating compounds with features distinct from the conventional chromophore/side-chain ones. Some approaches, exemplified by G4-QuadScreen (https://chemopredictionsuite.com/, last accessed 14 July 2024), have used a multi-targeting approach against several G4s [111]. This has been successful in identifying 62 hits from a library of 631,475 natural product compounds collected from large compound databases such as ZINC (https://zinc15.docking.org/, last accessed 14 July 2024). The final three lead compounds have cell growth-inhibitory activities (IC_50_ values) against a small panel of cancer cell lines of 6–30 µM. Tellingly, the G4 and cellular selectivity of these were not found to be obviously related.

b. **It is possible to unequivocally identify the complete set of G4-containing genes in a cell type** [32,112,113,114,115]. However, we are very far at present from identifying, validating, and determining the tertiary folds of the totality of G4s encoded within the promoters of all these genes. The scale of this challenge is again highlighted by the very small number of promoter gene G4s for which structural data are currently available and which have been targets for structure-based design (see for example references [116,117,118]);
c. Targeting a single G4 may be therapeutically sufficient in some cancers that have a single dominant driver gene, e.g., *c-KIT* in gastro-intestinal tumors (GIST), especially in early-stage disease [119]. Liposarcomas also fall into this category since they are characterized by dysregulation of the *MDM2* gene, which contains a G4 region in its promoter [120] and which can be successfully targeted by the G4 ligand QN-302 [121]. On the other hand, complex hard-to-treat cancers such as pancreatic cancer (PDAC) involve the dis-regulation of many genes and their pathways [122,123]—there are, therefore, very many G4s that are potential targets in these diseases.

PDAC continues to be the focus of the G4 targeting project at The School of Pharmacy and is introduced at this point in this paper. We then discuss the discovery and development of the novel small molecule compound QN-302 in the light of the challenges posed in the earlier sections, and we describe the approach that we have developed to circumvent the current limitations of G4 targeting methods.

## 4. The Discovery and Optimization of Substituted Naphthalene Diimides Targeting G4s

### 4.1. An Overview of Pancreatic Cancer

Pancreatic ductal adenocarcinoma (PDAC) remains one of the most intractable of human cancers, with a continuing high mortality and low long-term survival rate, combined with resistance to effective therapeutic approaches [124,125,126,127]. Small-molecule therapy in PDAC has not to date made a major difference to patient outcomes [128]. The widely used standard-of-care nucleoside drug gemcitabine [129,130] is only palliative and extends the life span in patients with advanced, metastatic PDAC disease by a few months at most. Other more aggressive therapies, for example the FOLFIRINOX combination (5-fluorouracil, irinotecan, oxaliplatin, and folinic acid), can only be applied to that fraction of patients able to withstand the major side-effects of the cytotoxic components [131]. PDAC is characterized by genetic complexity (see for example references [122,132]), even though mutations in four genes (*KRAS*, *CDKN2A*, *TP53*, and *SMAD4*) have been consistently described as major players in PDAC initiation. Many studies have identified large-scale gene expression changes, with a further >2000 genes contributing to PDAC metastasis and progression. Successful therapeutic approaches to PDAC are thus unlikely to be based on targeting single individual genes or their expressed proteins.

### 4.2. Early-Generation Naphthalene Diimides

The realization that the number of pendant groups on an appropriate planar heteroaromatic platform can play a critical role in G4 affinity and specificity was initially demonstrated for the BRACO-19 molecule [89,90], with its three substituent groups. Since all G4s, whatever their topology, have at least more than two grooves, albeit of varying geometries depending on the G4 topology, it was logical to consider enhancing the generic G4 specificity with three or four pendant groups. Several naphthalene imide and diimide compounds had been previously shown to bind by intercalation to duplex DNA and one, amonafide (5-amino-2-[2-(dimethylamino) ethyl]benzo[de]isoquinoline-1,3-dione), with a single cationic side chain, has been in clinical trials against several cancer types, although its severe toxicity has precluded its further development [133].

It was hypothesized, from qualitative molecular modelling using the human intramolecular telomeric G4 crystal structure [134], that the naphthalenediimde (ND) core moiety could effectively π–π stack onto a terminal G-quartet in typical G4s by virtue of its delocalized electron system. ND core geometry and its ability to stack on G4 ends would enable the delivery of three of four substituent sidechains into the grooves of G4s. These side chains would optimally have a terminal cationic character, which would enhance their binding to G4s via electrostatic interactions with the anionic phosphate-lined groove walls, whereas duplex DNA (and RNA) binding would not be feasible due to steric hindrance from the multiple side chains. An initial library of 26 tri- and tetra-substituted ND compounds was synthesized from the inexpensive commercially available starting compound 1,4,5,8-naphthalene tetracarboxylic dianhydride [135]. Compounds having, for example, four acyclic sidechains terminating in dimethylamine or pyrrolidine groups, were found to strongly stabilize a human telomeric G4 in solution, as measured by a FRET (fluorescence resonance energy transfer) assay. The G4 stabilizing ability of these compounds was broadly correlated with high potency in 96 h SRB (Sulforhodamine B) cell-growth inhibition assays, with optimal IC_50_ values of 5–10 nM. Active compounds also displayed moderate potency in a modified telomerase TRAP (telomerase repeat amplification protocol) assay. Mechanistic studies suggested that telomere uncapping was just one aspect of their mode of action, and that the transcriptional inhibition and induction of chromosome instability were also possibly involved [136].

A subsequent analysis of three tetra-substituted NDs with all four side chains terminating in N-methyl piperazine groups examined their anti-proliferative activity in a small panel of seven cancer cell lines, together with their G4 binding and telomerase inhibitory activity [137]. This showed that the two pancreatic cancer lines, PANC-1 and MIA-PaCa2, were the most sensitive, and that G4 stabilization was necessary but, by itself, was an insufficient indicator of biological activity. The most active of the three was subsequently examined in the MIA-PaCa2 and HPAC tumor xenograft models, in which ca 50% and 30% decreases in tumor volume were observed with a dose of 3 mg/kg three times weekly [138]. Telomerase inhibition was also observed in vitro and in vivo, although it was recognized that this was unlikely to be the sole mode of action.

Crystallographic studies of G4 complexes with two tetrasubstituted NDs played a role in the optimization of the ND sidechains, using a small, focused library of nine ND derivatives with varying cyclic and cationic end groups and side chain lengths [139]. This resulted in the identification of the lead compound MM41 (Figure 1: 4,9-Bis((3-(4methylpiperazin-1-yl)propyl)amino)-2,7-bis(3-morpholinopropyl) benzo[lmn] [3,8]phenanthroline-1,3,6,8(2H,7H)-tetraone), which was then chosen for detailed mechanistic study. This compound, with two of the N-methyl-piperazine groups replaced by less basic morpholino ones, was consistently the most potent in a small panel of cancer cell lines (Table 3). It was also notable that, within the panel, MM41 was most active against a lung (A549) and a pancreatic cancer line (MIA-PaCa2), whereas it was some 30–50-fold less active against two renal cancer lines (786-0 and RCC4). Interestingly, although MM41 strongly stabilized a telomeric G4 as judged by a FRET assay, the increases in G4 melting behavior (ΔT_m_ values) were comparable to those observed with several other less potent ND derivatives. MM41 also showed significant activity in a mouse xenograft model of PDAC [140]. Twice-weekly 15 mg/kg doses resulted in a ca 80% tumor growth decrease. Two animals in a group of tumor-bearing animals survived tumor-free after 279 days. This second encouraging indication of potent in vivo activity for an ND compound [138] led to a decision that further optimization was warranted.

### 4.3. The Development of the Phenotypic Approach to Later-Generation ND Compounds

The knowledge that several tri- and tetra-substituted G4-binding NDs display selective potency towards pancreatic cancer cell lines, combined with activity against in vivo models of human pancreatic cancer, gave further support to the suggestion that G4-containing genes may be involved in the disease, and could be targets for the NDs. Inspired by the demonstration [45] that the expression of a cancer gene (*MYC*) can be down regulated by a G4 ligand led to an emphasis on promoter G4 targeting by NDs. This decision was supported by subsequent data showing that transcriptional downregulation was predominantly observed for genes enriched in promoter G4s, although this does not differentiate between these genes being direct or indirect targets.

The concept has been taken further by examining ligand binding to a small panel of G4 targets (typically 5–10 G4 promoter sequences). In some instances, selectivity was found, but we were cognizant of the limitations both of panels containing small numbers of G4s and of attempts at the structure-based design and optimization of further NDs (see Section 3 above). A substantial modification of the MM41 molecule was made by decreasing the number of side chains, each still with a formal cationic charge, and thus also reducing its molecular weight. This resulted in the third-generation compound CM03 (Figure 1), with just three pendant side chains [64]. The design concept was supported by the crystal structures of several complexes between MM41 and a human intramolecular telomeric G4 [139,141], and were used as generic G4 structures. These crystal structures did reveal that the area of the ND chromophore did not fully cover all the surface of the stacked G-quartets and thus precluded all four side chains from simultaneously binding in the G4 grooves. Therefore, three side chains would suffice for effective binding. However, since the precise G4 targets of the NDs were not known at this time, the crystal structures and associated modelling, and the biophysical measures of G4 binding, could not fully define the optimal trisubstituted compound. Instead, cell-growth inhibitory data were again used as the primary filter—this is the phenotypic approach traditionally used in drug discovery [142]—which released the ND ligand optimization process from the limitations of not being able to optimize with respect to the real G4 target(s). The resulting compound, CM03, is similarly potent to MM41 in pancreatic cancer cell lines and in the MIA-PaCa2 in vivo xenograft model for PDAC, as well as in the therapeutically demanding in vivo genetic model for PDAC, the so-called KPC model [143].

Unsurprisingly, studies that have examined effects on multiple genes, initially using micro-array methods [139] and more recently using whole-genome RNA sequencing (RNA-seq), have revealed an altogether more complex picture than was assumed at the outset, with binding data obtained from a very small number of G4s. These later studies revealed that a typical G4-binding ND ligand can affect the transcription of many hundreds of genes—the trisubstituted ND compound CM03 down-regulates 2272 genes following a 24 h exposure in MIA-PaCa2 cells [140]. It is significant that this down-regulated gene set is enriched in G4 promoter sequences, whereas the upregulated gene set is not. The concept of targeting multiple G4s with NDs arose from this data analysis, which also showed that these down-regulated genes were frequently found in multiple cancer-related pathways. Some, such as the WNT/β-catenin pathway, are generic to several cancers, whereas others, such as the axon-guidance pathway, are specific to PDAC. Surprisingly some expected gene targets, notably *MYC*, were unaffected by CM03. It was concluded that the downregulation of multiple genes was likely to be an important factor in the anti-tumor activity of CM03 in animal models (and possibly also in humans).

### 4.4. The Current Lead Compound, the Experimental Drug QN-302

Any drug discovery campaign asks the constant question: can one further improve the current lead compound? The trisubstituted ND derivative CM03 was the starting-point for the attempt to overcome this challenge, which set out to improve its in vitro and in vivo biological potency by an order of magnitude, as well as ensuring as far as possible that off-target toxicity was not a constraint to future clinical development, all perhaps in concert with enhanced G4 affinity.

**Figure 2:**
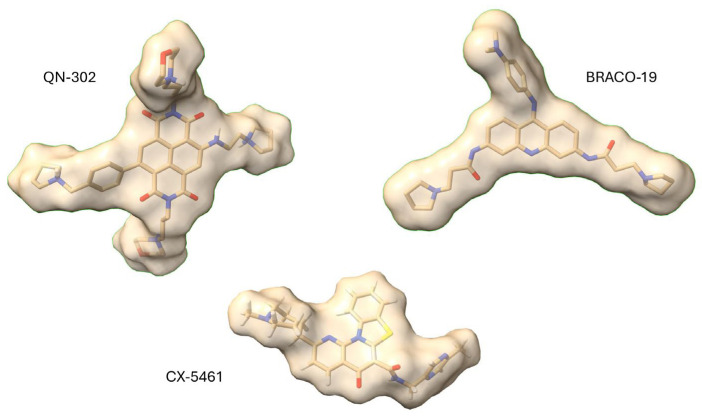
Surface representations of three well-established G4 binders, drawn to the same scale. The four side chains of QN-302 can be seen—three are oriented out of the plane of the page and the fourth, with the benzyl-pyrrolidine group, is seen on the left-hand side of the molecule. Two of the three side chains of BRACO-19 are in the plane of the page; these have similar dimensions to two of the QN-302 ones and would be expected to bind in G4 grooves in a similar mode.

A conservative approach was taken, with the three side chains of CM03 kept fixed and variations made at the position on the ND core where a fourth substituent had been in MM41 (Figure 1). A total of 84 compounds were synthesized and a short-list was selected using FRET to measure the quadruplex stabilization (resulting in G4 melting temperature ΔT_m_ values) for a small G4 panel together with a standard duplex DNA, and obtain cell-growth inhibition data (IC_50_ values, initially for a single PDAC cell line). The former enabled low-affinity compounds to be rejected but did not provide discrimination between compounds with greater G4 stabilization properties. On the other hand, the cellular assays enabled successive short-lists to be established of, firstly, nine compounds, and, finally, three (CM03, SOP1247, and QN-302: see Figure 1). Data for the long short-list of nine compounds were augmented by in vivo pharmacokinetic data, to ensure that the chosen candidate compounds would have sufficient in vivo bioavailability, together with data from receptor-binding assays, to minimize any off-target effects. The final choice was informed by data from a panel of PDAC cell lines (Table 4). QN-302 (initially termed SOP1812 [10]) was the outstanding and most active compound of the series, with anti-proliferative activity in most of the PDAC lines that was up to 10-fold greater than that of the other two compounds [144]. Thus, the anti-proliferative activities (IC_50_ values) for QN-302 are at single-digit nM levels. It also has a half-life (T_1/2_) in vivo of 37 h and there is no evidence of hERG or other receptor liabilities at or near therapeutic doses. Its superior activity was also observed in in vivo xenograft and KPC models for PDAC, with significant anti-tumor activity at a once-weekly dose of 1 mg/kg over four weeks, compared to the 10–15 mg/kg dosing required for CM03. Both CM03 and QN-302 are active in gemcitabine-resistant PDAC cell lines (Table 5), with the latter retaining its 10-fold superiority in potency [145,146]. Molecular modelling using the MM41-telomeric G4 crystal structures [139,141] has provided a plausible rationale for the superiority of QN-302. It is a consequence of the longer benzyl-pyrrolidine substituent being able to protrude deep into a groove and stacking onto an adjacent G-quartet. However structural data on the most plausible G4 targets (see below) are not currently available, so modelling is most useful in providing qualitative support to the phenotypic data (Figure 2). It remains the case though that potent G4 stabilization is an absolute requirement in this series of NDs: those members of the 84-compound library with low G4 ΔT_m_ values also had low cellular potency.

The transcriptome data obtained after 24 h exposure of MIA-PaCa2 PDAC cells to QN-302 [10] have been further analyzed [144]. Twelve genes have been identified to have the most significant downregulation in expression (Table 6). Almost all play a role in human pancreatic cancer, seen by mapping them on to their expression levels in pancreatic cancer patients, using data available from the Human Protein Atlas (https://www.proteinatlas.org/, last accessed on 12 July 2024). It is notable that QN-302 does mostly affect the same pathways as CM03 and SOP1247 [144]. However, QN-302 has a greater effect on genes distinct from those sensitive to these two compounds (Table 6). Here, we briefly highlight two examples of well-characterized genes and their encoded proteins, which are detailed elsewhere [144]. To date, the only G4s examined in this list of G4-rich genes are those in the *S100P* gene.

- The *GLI1* gene encodes the major transcription factor GLI1 in the Hedgehog pathway and its expression is down-regulated to a greater extent by QN-302 than by the other two compounds, which mostly affect the expression of the *GLI4* gene, which is of lesser therapeutic significance. The GLI1 protein is significantly upregulated in human PDAC and has been studied as a potential therapeutic target since its upregulation promotes cell migration and metastasis;
- The *S100P* gene is frequently upregulated in PDAC, and both the gene and the S100P protein have been considered as plausible therapeutic targets and biomarkers in PDAC, since cancer cell apoptosis and anti-tumor activity are consequences of its targeting and downregulation ([147] and references therein). A plausible G4 sequence in the promoter of the *S100P* gene has been identified [147], 48 nucleotides upstream from the transcription start site. This sequence forms a stable G4 structure under physiological K+ conditions and is further stabilized by QN-302.

P is the calculated probability of each fold change. High/Low refers to human pancreatic cancer patient data taken from the human protein atlas, showing numbers of patients with high and low expression of each gene; P_protein_ is the calculated associated probability. PQS is the number of calculated putative G-quadruplexes in each gene, and PPQS represents the number in each promoter region. ACT indicates whether the gene/protein has been studied as an anticancer target, possibly with small-molecule inhibitors. VACT indicates whether it has been validated as an anticancer target. Limited in vivo data are available on the expression of these genes following in vivo exposure to QN-302. However, q-PCR data on the *S100P*, *MAPK11*, *CX3CL1*, and *PRDM16* genes in an in vivo pancreatic cancer model all show significant downregulation following QN-302 treatment [10,148] (unpublished observations). We have previously reported [148] that the *S100P* gene is upregulated in a sample set of poorly differentiated human pancreatic cancers. *CX3CL1*, *CLIC3*, and *MAPK11* are also significantly upregulated in these tumors (*p* < 0.05). However, the tumor sample set is too small for firm conclusions to be made on the wider prevalence of the gene set in these samples.

Notes

1. *S100P* codes for a Ca-binding protein involved in migration and metastasis and, especially in pancreatic cancer, binds to and inactivates p53.
2. *CX3CL1* codes for a chemokine involved in pancreatic cancer cell migration and viability.
3. *CLIC3* codes for a chloride ion channel associated with metastasis in pancreatic cancer.
4. *NTN4* codes for a laminin family member that inhibits senescence.
5. *SLC19A1* codes for the folate transporter gene, up regulated in several cancers.
6. *KRT16* codes for a keratin associated with cancer cell motility and metastasis.
7. *PRDM16* codes for a transcription factor that suppresses TGF-β signaling.
8. *RTN4R* codes for a protein that regulates AKT signaling and enhances cancer cell proliferation.
9. *GLI1* codes for a transcription factor that is a key effector of the Hedgehog pathway and an established anticancer target, notably in PDAC.
10. *MAPK11* codes for a mitogen-activated protein kinase involved in pathway regulation and cancer cell proliferation.
11. *HSPA1A* is a heat shock gene encoding for the HSP70 protein, associated with 20 different cancers.
12. *GPRC5B* codes for a G protein-coupled receptor class C member involved in extracellular glucose sensing and glucose metabolism. It is upregulated in several cancers including pancreatic cancer. It is regulated by the transcription factor RUNX1.

We suggest that the enhanced downregulation of this group of genes, several of which are known to be significant in PDAC (Table 6), may be a consequence of selectivity at the G4 level. However, at present, this must remain speculative in the absence of data as to which ones form stable G4s and which play a significant direct or indirect role in the cellular and in vivo potency of QN-302. It is of some future interest that several members of this 12-gene group are involved in other cancer types, suggesting that QN-302 may have potentially useful anti-cancer activity beyond PDAC (see Section 5 below).

### 4.5. QN-302 in the Clinic

The experimental anti-tumor activity of QN-302 in pancreatic and other cancers, together with its favorable pharmacological and chemical profile, has led to its out-licensing by UCL Business to Qualigen Therapeutics Inc as a novel cancer therapeutic agent, for pre-clinical and clinical development. The licensing occurred in January 2022. The drug was granted Orphan Drug status for PDAC by the Food and Drug Administration (FDA) one year later. This took into account the existing pre-clinical data, including the findings that toxicity was not observed at therapeutic doses in any of the animal models used for PDAC. Subsequent toxicological, scale-up syntheses and formulation studies led in mid-2023 to an Investigational New Drug (IND) proposal to the FDA. Consent was granted in summer 2024 for a Phase 1 dose escalation clinical trial on advanced solid tumors, including PDAC. The trial is currently ongoing and started with an initial dose of 1.67 mg once a week over four weeks, administered intravenously. The limited assessments to date do suggest that (1) the drug is well tolerated, and no significant dose-limiting toxicity has been observed at low doses, with patients reporting a good quality of life. (2) Several PDAC patients showed no disease progression for periods of up to four months [9].

## 5. Conclusions

There are currently two experimental G4-binding drugs undergoing clinical assessment, QN-302 and the fluoroquinolone-based compound CX-5461 (Figure 1) (Pidnarulex) [6,7,8,148]. The latter, by contrast with QN-302, shows selectivity for BRCA1/2-deficient cells [6,7,8,148] and cancers, a selectivity that also appears to have been found by phenotypic selection. Although its G4 targets have not been disclosed, it also functions as a RNA polymerase I poison and a topoisomerase inhibitor [149,150], suggesting that it is a distinct multi-targeting agent, with consequent advantages in the treatment of certain repair-deficient complex cancers. The results of the Phase 1 trial with Pidnarulex [7] in a group of 46 patients with advanced solid tumors, emphasizing those with defective DNA repair pathways, showed 14% responses, notably in those individuals with defective homologous recombination. This clinical proof of concept outcome supports the rationale of the drug’s G4-related mechanism of action and paves the way for further clinical trials, even though dose-limiting phototoxicity was observed in the patient group. This effect appears to be confined to the fluoroquinolone chemotype of Pidnarulex, since a control in vitro phototoxicity assay with the G4 ligand pyridostatin did not show the same effect. This result is significant in demonstrating that phototoxicity is not a general feature of G4 ligands.

Another example of the possible future development of QN-302 beyond PDAC comes from preliminary screening data with a panel of prostate cancer cell lines [151], in a further extension of the phenotypic approach to discovering further cancer categories sensitive to this drug. This study has shown selective and low single-digit activity for QN-302 in the PC-3 cell line (Table 7), which is a model for castration-resistant prostate cancer when antiandrogen hormone therapies are no longer effective. This effect is seen in Table 7 for the two clinically used drugs Abiraterone and Enzalutamide, with both having high IC_50_ values in the PC-3 line. An initial therapeutic study with the PC-3 xenograft model has shown statistically significant (*p* = 0.0008) anti-tumor activity in this model, with 1 mg/kg twice-weekly dosing.

**Figure 3:**
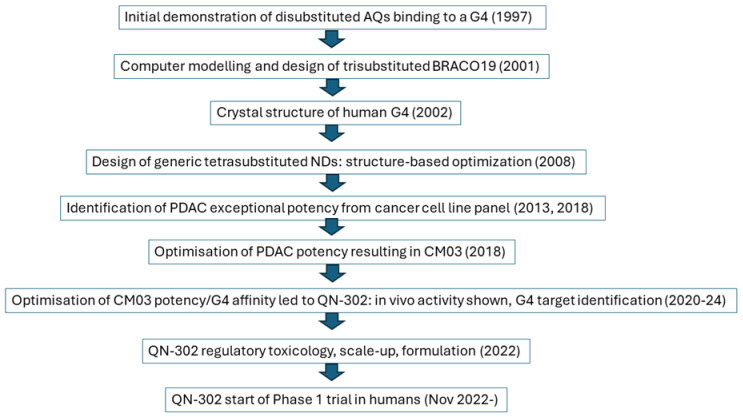
Flowchart of the history and phenotypic/structure-guided development of the experimental drug QN-302.

The phenotype-driven strategy presented here puts a disease (in this case pancreatic cancer) at the center of the discovery and optimization process for a discrete class of G4 ligands based on the ND core (Figure 3). Thus, the phenotypic strategy has been successful in the case of QN-302 and was born out of the necessity of circumventing the current restricted knowledge of G4 structure and function. Even so, the G4 paradigm has been an essential aspect of this approach in enabling effective lead chemotypes to be selected. G4 binding is the primary screen that has established the ND chemotype as appropriate for development but, as discussed here, is not currently able to, by itself, define an optimal lead compound. It is hoped that phenotypic screening in other G4 ligand projects will help in the identification of new classes of G4-targeted drug-like compounds for the treatment of other challenging cancers with unmet clinical need, even as we learn more about the G4 story.

## Figures and Tables

**Table 1:** Human promoter DNA G4s for which molecular structures have been determined, taken from the Protein Data Bank (PDB), indicating the method used for structure determination.

Gene	PDB ID	Method	Gene	PDB ID	Method
*MYC*	1XAV	NMR	*PARP1*	6AC7	NMR
*BCL2*	2F8U	NMR	*MYC*	6AU4	X-ray
*c-KIT1*	2KQG	NMR	*WNT*	6L8M	NMR
*c-KIT2*	2KYO	NMR	*K-RAS*	6SUU	NMR
*hTERT*	2KZD, 2KZE	NMR	*PDGFR*	6V0L	NMR
*MYC*	2LBR	NMR	*K-RAS*	6WCK	X-ray
*VEGF*	2M27	NMR	*MYC*	6ZL2, 6ZL3	NMR
*c-KIT1*	3QXR	X-ray	*BCL-2*	6ZX6, 6ZX7	NMR
*B-RAF*	4H29	X-ray	*MYC*	7KBV, 7KBW, 7KBX	NMR
*c-KIT1*	4WO2, 4WO3	X-ray	*VEGFR*	7XFV	NMR
*K-RAS*	512V	NMR	*RET*	7YS5	NMR
*VEGF*	5ZEV	NMR	*EGFR*	8JFQ	NMR

**Table 2:** Human promoter DNA ligand-G4 complexes for which molecular structures have been determined, taken from the Protein Data Bank (PDB), indicating the method used for structure determination.

Target G4	Ligand	Method	PDB ID
Human telomeric G4	BRACO-19	X-ray	3CE5
Human telomeric G4	Telomestatin	NMR	2MB3
Human telomeric G4	Berberine derivative	X-ray	72UR
G4-duplex hybrid	Phen-DC3	NMR	8ABD
*RET* promoter G4	Trioxacarcin A, cov. bound	NMR	8GP7
*KRAS* promoter G4	Coptisine	NMR	7X8O
Human telomeric G4	Phen-DC3	NMR	7Z9L
G4-duplex hybrid	Pyridostatin	NMR	7X2Z, 7X3A
G4-duplex hybrid	SYUIQ-5	NMR	7PNG
*MYC* promoter G4	Berberine	NMR	7N7E
*PDGFR* promoter G4	Berberine	NMR	7MSV
*MYC* promoter G4	NSC85697	NMR	7KBX
*VEGF* promoter G4	Platinum complex	NMR	6LNZ
*MYC* promoter G4	DC-34	NMR	5W77
*MYC* promoter G4	DAOTA-M2	NMR	5LIG
Human telomeric G4	MM41	X-ray	4DA3, 3UYH
Human telomeric G4	BMSG-SH-3	X-ray	4DAQ

**Table 3:** Cell-growth inhibition data (IC_50_ values in nM) for the tetrasubstituted ND compound MM41 [139] in a panel of cancer cell lines and a normal fibroblast line (WI-38), using a 96 h SRB assay.

Cell Line	IC_50_
A549	<0.010 ± 0.005
RCC4	0.56 ± 0.05
MIA-PaCa2	0.01 ± 0.01
786-0	0.32 ± 0.01
MCF-7	0.070 ± 0.007
WI-38	0.23 ± 0.01

**Table 4:** Cell growth inhibition data (IC_50_ values in nM) for three ND compounds in four PDAC cell lines, from 96 h SRB assays. Esds are ±0.1–0.5 nM [145]. Data for QN-302 is highlighted in yellow.

Cell Line	CM03	SOP1247	QN-302
MIA-PaCa2	9.0	13.8	**1.3**
PANC-1	15.6	15.7	**1.4**
CAPAN-1	26.5	38.8	**5.9**
Bx-PC3	15.5	20.5	**2.6**

**Table 5:** Cell growth inhibition data (IC_50_ values, in nM) from 96 h SRB assays, for two ND compounds, together with gemcitabine and CX-5461, in two pairs of parental and gemcitabine-resistant PDAC cell lines [147,148]. Data for the experimental drug QN-302 are highlighted in yellow.

Compound	MIA-PaCa2 Parental	MIA-PaCa2 GemResist	PANC-1 Parental	PANC-1 GemResist
Gemcitabine	6.5 ± 0.7	11,055.7 ± 540.0	23.3 ± 8.4	28,750.9 ± 6121.3
CM03	13.0 ± 8.4	14.9 ± 8.3	10.4 ± 1.2	15.5 ± 1.8
CX-5461	90.3 ± 30.7	88.7 ± 22.0	32.9 ± 7.6	58.8 ± 13.8
**QN-302**	**2.6 ± 1.0**	**3.8 ± 1.2**	**2.3 ± 0.4**	**3.3 ± 0.7**

**Table 6:** Detailed RNA-seq expression data for the top 12 cancer-related genes showing down-regulated expression. All have large and, for the most part, statistically significant fold changes in mRNA expression, following 24 h QN-302 treatment of MIA-PaCa2 cells. The RNA-seq data set is available in the GEO public functional genomics data repository (https://www.ncbi.nlm.nih.gov/geo/, last accessed on 3 May 2024), and is listed as GSE151741 for QN-302.

Gene	Log_2_FC	FC	P	High/ Low	P_protein_	PQS	PPQS	ACT	VACT
*S100P*	−3.23	9.4	0.08	124/52	0.0002	60	8	Y	Y
*CX3CL1*	−2.91	7.5	0.04	62/114	0.026	5	2	Y	Y
*CLIC3*	−2.77	6.8	0.07	140/36	0.008	6	3	?	Y
*NTN4*	−2.46	5.5	0.015	129/41	0.0001	13	4	N	Y
*SLC19A1*	−2.27	4.8	<<0.0001	138/38	0.29	38	4	?	?
*KRT16*	−1.98	3.9	0.0016	140/36	0.00001	2	1	N	Y
*PRDM16*	−1.91	3.8	<<0.0001	140/36	0.052	260	5	?	?
*RTN4R*	−1.87	3.7	<<0.0001	43/123	0.012	28	3	N	Y
*GLI1*	−1.84	3.6	0.011	53/133	0.085	15	4	Y	Y
*MAPK11*	−1.72	3.3	<<0.0001	85/91	0.005	18	6	Y	Y
*HSPA1A*	−1.15	2.2	0.0001	65/111	0.008	3	1	Y	Y
*GPRC5B*	−1.14	2.1	0.0025	125/51	0.028	4	1	N	N

**Table 7:** Cell viability activity in a panel of prostate cancer cell lines for two ND compounds, the clinically approved antiandrogen drugs abiraterone and enzalutamide. Evaluations were undertaken using an XTT (Roche) proliferation assay. Calculated IC_50_ values are given in nM. Esds vary from ca 0.4 to ca 20 nM, depending on the size of the IC_50_ value. Data for the drug QN-302 in the PC-3 cell line is highlighted.

Prostate Cancer Lines	CM03	QN-302	Abiraterone	Enzalutamide
PC-3	94	**3**	4820	5350
DU145	113	32	N/A	N/A
LNCaP	394	247	3860	4820
VCaP	135	68	N/A	N/A
22RV1	90	90	N/A	N/A

## Data Availability

The RNA-seq data set is available in the GEO public functional genomics data repository (https://www.ncbi.nlm.nih.gov/geo/, last accessed on 3 May 2024), and is listed as GSE151741 for QN-302.
